# Supplementary material for: Learning curve and surgical outcome of robotic assisted colorectal surgery with ERAS program
Source: Sci Rep. 2022 Nov 29;12:20566. doi: 10.1038/s41598-022-24665-w (PMC9709162; doi:10.1038/s41598-022-24665-w)
Supplement: Supplementary file 1 — Supplementary Information 1. [file 41598_2022_24665_MOESM1_ESM.zip › LSC Raw data-Ñ[▒K/104/H_ñw╝╨░O▒Kñσ.pdf]

正式摘要(電子簽章：000000203422566)		
W52 / W52 ☐英文標題(注意:無英文姓名-請補病人基本檔!)	☐【讀取】憑證	

臺中榮民總醫院

出院病歷摘要

轉入號： 轉出號：

[redacted]

(8)流水編號	
(9)入院日期	109/11/11 (2020) 直腸外科 W52 - 111
(10)轉科日期	
(11)出院日期	109/11/17 (2020) 住院天數計 6 日

診斷

- 入院:rectal tumor, upper third
- 出院:rectal tumor, upper third, pT3N1bN0 s/p laparoscopic LAR on 20201112

主訴

- blood tinged stool was noted with easily diarrhea for 3-4 months

病史

- This is a 45 y/o man who presented with blood tinged stool was noted with easily diarrhea for 3-4 months. He first went to LMD where upper rectum tumor was noted. He then went to our CRS OPD. This time, he is admitted for operation.

體檢發現

-
  - General appearance :
    - easy looking , conscious clear , without cardiopulmonary distress
    - Vital signs自動帶入date 體溫(BT) 脈搏(P) 呼吸(R) 血壓(BP) 身高(BH) 體重(BW) BMI
      20201111 36.1℃ 84次/分 18次/分 111/82mmHg 160.2cm 59kg 23.0
  - HEENT :
    - Eyes: no anemic conjunctiva, no icteric sclera, normal EOM
    - ENT: no otorrhea, no rhinorrhea, no erythematous mucosa, no injected tonsil, no deformity
  - Neck: supple, freely movable, trachea midline,no deformity, no engorged jugular vein , no palpable thyroid or lymph node enlargement
  - Chest:
    - Inspection: Grossly normal, symmetric and fully expansion, no accessory muscle used, no operation scar, no spider angiomata, no gynecomastia
    - Palpation: no palpable mass or nodule, no subcutaneous emphysema, no tenderness
    - Ausculation: clear breathing sound
  - Heart:
    - Percussion: normal heart size
    - Ausculation: regular heart beat, normal S1, S2, no gallop, no murmur
  - Abdomen:
    - Inspection: in
      - flat contour, symetrically, no scar, no stria, no dilated veins, no rashes
    - Ausculation: normoactive bowel sound, no abdominal bruits
    - palpation: soft, no muscle guarding or rigidity, no local tenderness, no rebounding pain, no palpable mass, no palpable pulsation
    - Percussion: no shifting dulllness, no hepatomegaly, no splenomagaly
  -

Peripheral pulsation :	CA	BA	RA	FA	PA	PTA	DPA
Right side :	++	++	++	++	++	++	++/+++

- | Left side :|++|++|++|++|++| ++ |++/+++|
  - Genito-anus: no tenderness or palpable mass lesion or rectal shelf, normal external genitalia, normal anal tone, brown stool

CRS CONDITION

-
  - Lung

手術日期及方法

- 手術日期及方法20201112 1. Laparoscopic anterior resection, end-to-end anastomosis, Endo-GIA 60-4.1mm, CDH 29mm.
  Bilateral doughnut: intact, air tight test: done, no air leak.
  2. High ligation (+) above the left colic artery.
  3. Cut margin: proximal 5.5 cm, distal 2 cm.
  4. Take down of splenic flexure: (-).
  5. No liver metastasis or carcinomatosis was noted
  6. No drain placement

住院治療經過

- After admission, operation was done on 11/12. Try clear liquid diet on 11/12. Try semiliquid diet on 11/14. Due to relative stable condition, MBD was permitted and OPD f/u is scheudled for further treatment.

合併症

一般檢查

特殊檢查

病理報告

其它

出院時情況

- 15.計劃性出院

出院指示

藥名	劑量	劑量單位	用法	頻次	數量	數量單位	現況	天數	單位
Ultracet tab	1	TAB	PO	Q6H	28	TAB	TH	7	天
Cap Trand 250mg	1	CAP	PO	TID	21	CAP	TH	7	天
Colchicine tab 0.5mg	1	TAB	PO	BID	14	TAB	TH	7	天
Ulstop tab 20mg	1	TAB	PO	BID	14	TAB	TH	7	天

出院計劃

主治醫師_2062F 蔣鋒帆_ / 住院醫師_2310K 陳瓊夫_

正式摘要(**電子簽章：000000203422566)**

W52 / W52

doctorRank:B
